# Molecular Mechanism of Action of Endocrine-Disrupting Chemicals on the Respiratory System

**DOI:** 10.3390/ijms252312540

**Published:** 2024-11-22

**Authors:** Francesco Molinari, Gianluca Antonio Franco, Nicla Tranchida, Rosanna Di Paola, Marika Cordaro

**Affiliations:** 1 Department of Veterinary Sciences, University of Messina, Viale SS Annunziata, 98168 Messina, Italy; francesco.molinari@studenti.unime.it (F.M.); gianluca.franco@studenti.unime.it (G.A.F.); 2 Department of Chemical, Biological, Pharmaceutical and Environmental Sciences, University of Messina, Viale F. Stagno D’Alcontres 31, 98166 Messina, Italy; nicla.tranchida@studenti.unime.it; 3 Department of Biomedical, Dental and Morphological and Functional Imaging, University of Messina, Via Consolare Valeria, 98125 Messina, Italy; cordarom@unime.it

**Keywords:** endocrine disruptors, lung health, asthma, COPD, pulmonary fibrosis, oxidative stress, inflammation, blood–air barrier

## Abstract

Endocrine-disrupting chemicals (EDCs) are a growing health hazard for humankind and respiratory health in particular. Such chemical compounds are present in the environment and food and may interfere with physiological processes through interference with functions of the endocrine system, making humans more susceptible to various types of diseases. This review aims to discuss the effects of EDCs on the respiratory system. Exposure to EDCs during fetal development and adulthood increases susceptibility to respiratory diseases such as asthma, COPD, and pulmonary fibrosis. EDCs are both multiple and complex in the ways they can act. Indeed, these chemicals may induce oxidative stress, modify cell proliferation and differentiation, interfere with tissue repair, and modulate the inflammatory response. Moreover, EDCs may also break the integrity of the blood–air barrier, allowing noxious substances to penetrate into the lung and thus enhancing the opportunity for infection. In conclusion, the scientific evidence available tends to indicate that EDCs exposure is strongly linked to the initiation of respiratory disease. Further research will be important in discovering the underlying molecular mechanisms and devising preventive and therapeutic measures.

## 1. Introduction

Endocrine-disrupting chemicals (EDCs) are exogenous substances, natural or synthetic, that can interfere with the endocrine system of living organisms, altering their physiological functions [1]. In other words, these substances can mimic, block, or enhance the action of natural hormones, causing a wide range of adverse health effects. The classification of endocrine disruptors is complex due to their structural diversity and multiple mechanisms of action [2]. However, they can be grouped according to several characteristics (Table 1). Endocrine disruptors represent a pervasive threat to human health, infiltrating our bodies through numerous pathways [3]. Food, contaminated with pesticides or packaged in materials that leach chemicals, is a primary source of exposure [4]. Inhalation of polluted air, dermal contact with cosmetics, detergents, and treated fabrics, and occupational exposure in certain industries also contribute to this contamination [5]. Once inside the human body, these substances can disrupt the delicate endocrine system, leading to a wide array of health problems, including reduced fertility, neurological disorders, and cancer [6,7].

The lungs, with their vast exchange surface area and constant exposure to the external environment, are an organ that is particularly susceptible to the harmful effects of EDCs [15]. Their primary function, gas exchange, places them in direct contact with a wide variety of substances in the air, including EDCs. This prolonged and direct exposure makes them a prime target for these molecules [16]. The anatomical and functional complexity of the lungs makes them particularly vulnerable. The presence of a vast network of capillaries, in close contact with the pulmonary alveoli, favors the rapid uptake of EDCs [17]. Moreover, the cells lining the alveoli, the pneumocytes, express a variety of hormone receptors, making them sensitive to the action of EDCs that mimic or block the action of natural hormones. The effects of EDCs on the lungs are manifold and can occur in the short and long term [18]. In the short term, exposure to high concentrations of EDCs can cause airway irritation, coughing, wheezing and, in severe cases, bronchospasm [19]. In the long term, chronic exposure may contribute to the development of chronic respiratory diseases such as asthma and chronic obstructive pulmonary disease (COPD), and increase susceptibility to lung infections [20,21,22]. Some studies suggest that EDCs may also play a role in the development of lung cancer [23]. By interfering with DNA repair mechanisms and promoting chronic inflammatory processes, EDCs could promote malignant transformation of lung cells. Importantly, the vulnerability of lungs to EDCs is not limited to adulthood [24]. Fetal and childhood exposure to these contaminants may have long-term consequences on lung development, increasing the risk of developing respiratory diseases in adulthood [25]. This review provides a comprehensive overview of the molecular mechanisms underlying the adverse effects of endocrine-disrupting chemicals (EDCs) on respiratory health, with a particular focus on the role of inflammation and oxidative stress.

## 2. Effects on Lung Physiology

### 2.1. Fetal and Neonatal Development

The ontogeny of the respiratory system, involving the airways and lungs, is a multifaceted process that commences during the embryonic stage at 4–7 weeks gestation and extends well into adolescence. This prolonged developmental trajectory renders the developing respiratory system susceptible to disruption by deleterious factors. Moreover, the repair mechanisms within immature lung tissue are less efficient than those in the mature lung, increasing its vulnerability to injury. Consequently, exposure to EDCs during fetal and neonatal life poses a serious threat to lung development, with potential repercussions on long-term respiratory health. The developing lung, a highly dynamic organ that is sensitive to environmental stimuli, is particularly vulnerable to the effects of EDCs. These chemical compounds can interfere with the delicate growth and maturation processes of the lung, altering cell signaling, gene expression, and epigenetics [26,27]. The detrimental effects of EDCs exposure on pulmonary development can manifest in various ways. One potential consequence is a reduction in the number of alveoli, the tiny air sacs responsible for gas exchange [17]. Abnormal vascular development, including the formation of a malformed network of blood vessels within the lungs, may also occur [28]. Furthermore, alterations in the extracellular matrix, the structural component that supports lung tissue, can further compromise respiratory function [29]. The long-term consequences of EDCs exposure during development can be far-reaching. Individuals exposed to EDCs may be at an increased risk for developing a variety of respiratory diseases, such as chronic obstructive pulmonary disease (COPD), asthma, lung restriction, and lung cancer [30,31,32]. Numerous studies have highlighted the negative effects of several EDCs on lung development, including bisphenol A, phthalates, and pesticides [31,33,34].

### 2.2. Respiratory Function

One mechanism whereby EDCs could damage lung function is by inducing chronic inflammation at the alveolar level. D’Amico et al. demonstrated that the chronic inflammation may be dominated by a cellular infiltrate with the production of inflammatory mediators, leading to the eventual development of pulmonary fibrosis characterized by thickening of alveolar basement membranes, which decreases lung compliance [17]. Moreover, EDCs can interfere with the synthesis and release of surfactants important for the maintenance of alveolar stability and prevention of atelectasis [21]. The increased airway resistance is another important mechanism of action of EDCs. Exposure to such chemicals can induce bronchoconstriction, hyperplasia of mucous glands, and edema of the bronchial mucosa; this results in airway obstruction and subsequent respiratory distress [35]. The lungs are also significantly affected because of the induced changes in alveolar–capillary gas exchange. Alveolar damage and thickening of the basement membrane reduce the exchange surface and the diffusion of respiratory gases, causing impairment in tissue oxygenation [36]. EDCs could also affect the pulmonary vasculature, influencing the blood flow and the distribution of perfusion [23]. Finally, EDCs may modulate the airway inflammatory response by enhancing sensitivity to inflammatory mediators and promoting chronic inflammation [37]. This might contribute to chronic respiratory diseases like asthma and COPS.

### 2.3. Inflammation and Remodeling

The main impact of EDCs is the induction of oxidative stress interfering with the sensitive redox balance in the lung cells. A given imbalance may lead to increased production of ROS, highly toxic molecules that attack DNA, proteins, and lipids, provoking inflammation processes and cell death [38]. More specifically, EDCs interfere with various cell-signaling pathways involved in inflammation by modulating the activity of NF-κB and AP-1 transcription factors [17,39]. Chronic activation leads to the overproduction of inflammatory mediators and perpetuates the state of inflammation. Other mechanisms involve epigenetic modifications such as DNA methylation and histone modifications [27,40]. Epigenetic modifications of this sort bring about very long-lasting changes in gene expression that perpetuate chronic inflammation. EDCs finally act to damage mitochondria, which are the cellular organelles responsible for energy production [41]. Such mitochondrial dysfunction further promotes cellular damage through enhancing the production of injurious molecules, contributing to cell death [42].

### 2.4. Blood–Air Barrier

Notably, EDCs will affect respiratory health through interference with the permeability of the blood–air barrier, considered a key structural feature situated between the blood and lung tissue [43]. This would normally be impermeable to the greater part of substances, given the importance of its function: protection against infectious agents and toxins [22]. This barrier may weaken from the exposure to EDCs, becoming more permeable and enhancing the diffusion of harmful substances from the blood into the alveoli and vice versa, which is highly important for human health. It follows that the increased permeability of the blood–air barrier can stimulate resorption in the surroundings—toxic substances, air pollutants, and fine particles—increasing poisonous burdens both at the pulmonary and systemic levels [10]. Furthermore, this changed permeability allows the easy penetration of pathogens through lung tissue, enhancing vulnerability to respiratory infections. This is all the more relevant in people who already have chronic respiratory diseases, like asthma and COPD, in whom the integrity of the blood–air barrier has been compromised [22,44]. The molecular mechanisms of EDC actions on blood–air barrier permeability are complex and incompletely understood. However, it is assumed that EDCs may affect the endothelial cells and epithelial cells constituting the barrier through modifications in gene expression and cellular junctions [22]. EDCs may also induce an inflammatory response, resulting in further injury and enhanced permeability of the barrier. The alterations in the permeability of the blood–air barrier induced by EDCs have far-reaching consequences apart from respiratory diseases. Indeed, such alterations might be one of the contributory factors in the development of systemic conditions like cardiovascular disorders, neurodegenerative disorders, and autoimmune diseases [45,46,47]. In this regard, disturbance in blood–air barrier permeability represents an important mode by which EDCs can affect human health.

## 3. EDCs and Lung Diseases

### 3.1. Asthma and Allergies

**Figure 1:**
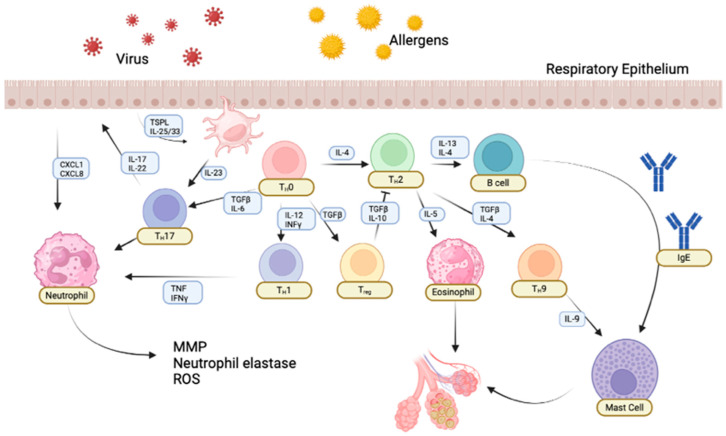
Graphical representation of the key steps in the development of asthma. Trigger exposure initiates immune activation and inflammation in the airways. Oxidative stress exacerbates inflammation, while transcriptional regulation amplifies the inflammatory response.

Epidemiological and experimental studies have, in the recent past, given a clearer picture of the association between the intake of EDCs and the development of respiratory diseases like asthma and allergies. Many epidemiological studies in populations with high exposure levels to EDCs revealed a significant increase in the incidence of asthma and allergic reactions, especially among children [48]. These observations have been reinforced by the available experimental studies in animal models, which indeed showed how even low-dose developmental exposure to EDCs disrupts the immune system maturation processes, predisposing individuals to the onset of allergic diseases [49]. The mechanisms of action for EDCs are apparently multifactorial and include but are not limited to modulation of the inflammatory response, disruption of epithelial barrier function, and disturbance in immune system regulation [23], as shown in Figure 1.

Recent studies have highlighted the potential for endocrine-disrupting chemicals (EDCs) to play a significant role in the development of allergic diseases. Bisphenol A (BPA) and Mono-n-butyl phthalate (MnBP) have emerged as two such compounds with concerning implications. Loffredo et al. [37] demonstrated that BPA exposure can interfere with epithelial homeostasis by disrupting estrogen-regulated signaling pathways. This disruption leads to increased epithelial proliferation and inflammation, both in vitro and in vivo. Notably, the systemic para-inflammatory response induced by BPA exposure was associated with enhanced allergic sensitization, suggesting a potential causal link. Quoc et al. investigated the effects of MnBP, a manganese-containing nanoparticle, on airway inflammation. Their findings revealed that MnBP can exacerbate neutrophilic inflammation in asthma by activating the autophagy pathway and promoting neutrophil extracellular trap (NET) formation [44]. Additionally, MnBP was shown to induce eosinophilic asthma by enhancing eosinophil activation, eicosanoid production, and CD4+ T-cell differentiation [50]. These studies collectively suggest that both BPA and MnBP can contribute to allergic sensitization and airway inflammation through distinct mechanisms. Furthermore, note that genetic and environmental factors interact in a very complex manner in influencing individual susceptibility to the development of allergic diseases in response to exposure to EDCs. Some studies have identified specific genetic polymorphisms that confer greater susceptibility to the effects of EDCs and thus predispose individuals to “priming” for the development of allergic diseases [51]. Moreover, it is believed that environmental factors such as atmospheric pollution, cigarette smoke, and allergens may interact with EDCs to potentiate their deleterious effects on respiratory health [23].

### 3.2. Chronic Obstructive Pulmonary Disease

**Figure 2:**
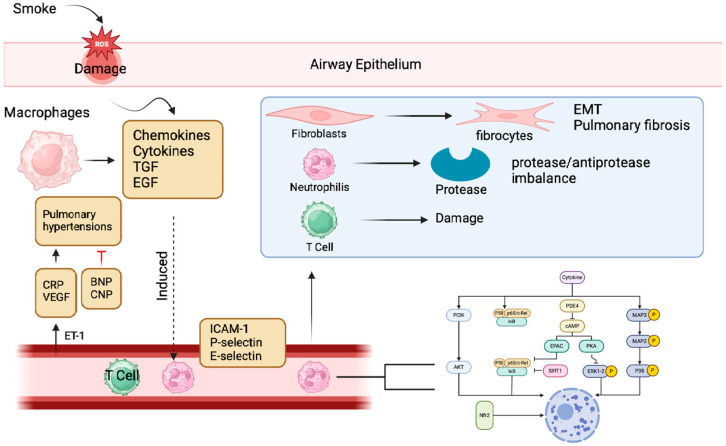
COPD pathogenesis involves oxidative stress, which injures lung cells, increases mucus, and triggers inflammation via redox-sensitive transcription factors. Cigarette smoke and infections cause oxidative stress, leading to the accumulation of inflammatory cells (neutrophils, CD8 T lymphocytes, macrophages) and reactive ROS, activated by NF-κB, p38MAPK, and PI3K pathways. Proteases contribute to tissue remodeling and ECM degradation. Inflammatory cytokines (LTB4, IL-8, TNF-α) worsen lung damage. PDE4 decreases cAMP, enhancing inflammation. Chronic inflammation raises EGFR and TGF-β1, promoting cell proliferation and mucus. TGF-β1 attracts immune cells, causing fibrosis. Endothelin-1 (ET-1) stimulates vascular muscle cells and VEGF synthesis. Natriuretic peptides (BNP and CNP) dilate blood vessels and reduce vascular resistance.

Although the exact mechanism is still not well known, several molecular mechanisms have been reported through which EDCs contribute to the characteristic decline in lung function seen in COPD (Figure 2).

Among these, the induction of oxidative stress is prominent [52]. Indeed, EDCs can induce the production of ROS and reactive nitrogen compounds, resulting in protein, lipid, and DNA damage in lung cells [53]. The chronic oxidative damage also leads to airway inflammation, destruction of lung parenchyma, and an accelerated decline in lung function [54]. Other major mechanisms involve the activation of proteases, which are enzymes responsible for the degradation of extracellular matrix proteins. EDCs may stimulate the production of proteases, such as neutrophil elastases that degrade elastin, a protein critical for maintaining the integrity of the alveoli [55]. The destruction of elastin contributes to emphysema, a major component of COPD [56]. Concomitantly, EDCs may inhibit the action of antiproteases, which are enzymes naturally antagonizing the action of proteases. Such a disturbance in the protease–antiprotease balance promotes further degradation of the extracellular matrix, leading to lung injury [57]. Furthermore, EDCs can impede the process of tissue repair via inhibition of epithelial cell proliferation and differentiation and alteration of tissue remodeling processes [58]. Finally, EDCs may potentiate other risk factors for COPD—for example, cigarette smoking, air pollution, and viral infection—resulting in a negative spiral that acts to hasten the progression of disease [23]. Recent studies have highlighted the detrimental effects of environmental pollutants on respiratory health, with a particular focus on their potential to contribute to chronic obstructive pulmonary disease (COPD). Several studies have implicated endocrine disruptors and particulate matter as key contributors to COPD-like phenotypes. Zhang et al. demonstrated that tributyltin (TBC), an endocrine disruptor with antiestrogenic properties, can induce COPD-like lung injury in mice when administered orally. This injury includes airway obstruction, inflammation, and pulmonary dysfunction. Importantly, estrogen treatment was found to alleviate TBC-induced lung damage, suggesting a potential therapeutic role for estrogen signaling in COPD [21]. Wang et al. investigated the long-term effects of respiratory cadmium (Cd) exposure in mice. Their findings revealed that chronic Cd exposure can lead to COPD-characteristic alveolar destruction, airway inflammation, and epithelial–mesenchymal transition (EMT). Furthermore, Cd exposure was associated with decreased lung function, further supporting its role in COPD development [59]. Li et al. conducted a study to assess the impact of long-term exposure to particulate matter 2.5 (PM2.5) on COPD pathogenesis. Their results showed that PM2.5 exposure can induce emphysema, decreased lung function, and both lung and systemic inflammation. These findings establish PM2.5 as a significant risk factor for COPD [60].

### 3.3. Lung Cancer

EDCs may induce pulmonary carcinogenesis through various direct and indirect molecular mechanisms [61]. Some directly damage DNA by interacting with DNA, inducing genetic mutations. Certain amounts of EDCs are capable of binding to DNA, forming additives which interfere with the replication and repair processes of DNA and thereby enhance the risk of accumulation of mutations and neoplastic transformation [62]. Moreover, EDCs are able to activate or inhibit enzymes that take part in DNA metabolism, including but not limited to DNA methyltransferases, thereby changing the DNA methylation pattern and further affecting gene expression [63]. Indirectly, carcinogenesis may also be encouraged by EDCs through the modulation of basic cellular processes: proliferation, survival, and angiogenesis. Some chemicals could have agonistic or antagonistic activity toward some hormones, which might interfere with cellular signaling and lead to uncontrolled proliferation. Also, EDCs can inhibit apoptosis, or programmed cell death, when the selective elimination of damaged cells has failed and these are allowed to survive and proliferate [64]. Angiogenesis is defined as a process involving the formation of new blood vessels. In particular, this process is an important step in supplying growing tumors with sufficient oxygen and nutrients [65]. Finally, EDCs may modulate chronic inflammation, a process that can contribute to genomic instability and tumor progression [66]. EDC-induced chronic inflammation may stimulate the production of reactive oxygen species, which in turn can cause DNA damage and gene mutation. In addition, chronic inflammation possibly promotes the activation of several factors that facilitate cell proliferation and survival [67].

### 3.4. Pulmonary Fibrosis

**Figure 3:**
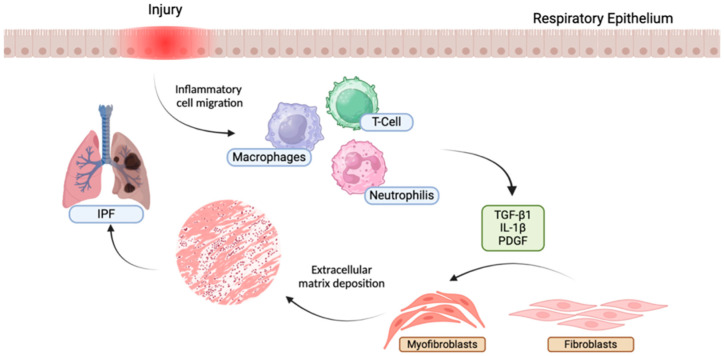
Overview of key wound healing stages leading to idiopathic pulmonary fibrosis (IPF): Epithelial cell injury causes inflammatory mediators’ secretion and platelet activation, increasing vessel permeability and leukocyte recruitment. These inflammatory cells release pro-fibrotic cytokines like TGF-β1, which activate and recruit fibroblasts, prompting their differentiation into myofibroblasts and the release of extracellular matrix (ECM) components for wound healing. In IPF, an abnormal wound repair response results in irreversible excessive scar tissue formation in the lungs.

Pulmonary fibrosis is a disease characterized by progressive scarring of lung tissue, leading to reduced respiratory capacity and ultimately death. Although the precise causes of pulmonary fibrosis are still not entirely clear, several studies suggest that EDCs may play an important role in the pathogenesis of this disease [17,68,69]. EDCs may influence the development of pulmonary fibrosis by acting on several fronts. First, EDCs may alter the proliferation and differentiation of fibroblasts, the cells responsible for the production of the extracellular matrix [70]. Fibroblasts are key cells in the repair of damaged tissues, but under pathological conditions such as fibrosis, they overproliferate and produce large amounts of extracellular matrix, leading to the formation of scar tissue. EDCs can stimulate the proliferation of fibroblasts and promote their differentiation into myofibroblasts, a subpopulation of fibroblasts characterized by increased contractile capacity and extracellular matrix production [71]. In addition, EDCs can influence extracellular matrix deposition by increasing the production of collagen and other extracellular matrix proteins. Excessive extracellular matrix deposition is a hallmark of pulmonary fibrosis and contributes to the rigid and inflexible scar tissue that characterizes this disease [70]. In addition to directly affecting fibroblasts, EDCs may act indirectly on lung fibrosis by modulating the inflammatory response and altering the tissue microenvironment [72]. Chronic inflammation is an important risk factor for pulmonary fibrosis, and EDCs may help perpetuate inflammation by promoting the production of pro-fibrotic cytokines and recruiting inflammatory cells to lung tissue as shown in Figure 3.

Recent studies have highlighted the detrimental effects of environmental pollutants on respiratory health, with a particular focus on their potential to induce toxicity and disrupt endocrine function. Vinclozolin, a fungicide, and particulate matter 2.5 (PM2.5) have emerged as two such compounds with concerning implications. D’amico et al. demonstrated that exposure to vinclozolin, especially when chronic, can induce significant alterations to the respiratory system. These alterations include increased inflammation, oxidative stress, and apoptosis. This study expands our understanding of vinclozolin’s effects beyond its known endocrine-disrupting properties, highlighting its potential toxicity to the respiratory system [17]. Zhuo et al. investigated the toxicity and endocrine-disrupting potential of PM2.5 from highway and industrial areas, considering factors such as the season and the presence of particulate polycyclic aromatic hydrocarbons (PAHs), polycyclic aromatic esters (PAEs), and heavy metals. Their findings revealed that the toxicity of PM2.5 was associated with PAHs and heavy metals, while its endocrine-disrupting potential was linked to di-ethylhexyl phthalate (DEHP), a widely used PAE. Additionally, the toxicity and endocrine-disrupting potential of PM2.5 were found to vary depending on the surrounding environment and season [15].

## 4. Conclusions

A growing body of evidence shows that endocrine disruptors are major contributors to respiratory health and have implications for the development and progression of various pulmonary diseases. While much has been learned regarding the possible molecular mechanisms by which EDCs can affect lung functions, some areas remain open to question. EDCA’s hindrance of respiratory diseases interaction cannot be fully explained without further research on identifying early biomarkers of EDC-induced lung damage. Further study is required to establish additional long-term effects of EDC exposure during fetal and infantile development. Meeting these challenges will require research in new directions, including the development of more realistic animal models that accurately reflect human EDC exposure, the use of “omics” approaches for the identification of novel biomarkers, and the analysis of interactions between multiple EDCs. In addition, such an approach must be interdisciplinary, with scientists from diverse disciplines participating in the in-depth study of various mechanisms by which EDCs may affect respiratory health. More research is needed in the long run for EDCs and pulmonary health to come up with appropriate preventative and therapeutic measures to protect respiratory health in populations. While the complete elimination of environmental pollutants is no easy task, a multifaceted approach does much in mitigating their impact. The exposure can be reduced by decreasing time in polluted areas, substituting products with environmentally friendly ones, and strengthening the body through proper nutrition rich in antioxidants and proper exercise. In the long term, reinforcement through policies and initiatives on clean energy and sustainable practices will contribute toward environmental health.

## Figures and Tables

**Table 1:** Classification of endocrine disruptors.

Origin	Mechanism of Action	Examples of Substances	Reference
Natural	Hormonal agonism	Phytoestrogens (soy isoflavones, lignans)	[8]
	Hormonal antagonism	Genistein (found in some legumes)	[9]
Synthetic	Hormonal agonism	Bisphenol A (BPA), phthalates (DEHP, DBP)	[10]
	Hormonal antagonism	DDT, some organochlorine pesticides	[11]
	Disruption of hormone synthesis	Dioxins, polychlorinated biphenyls (PCBs)	[12]
	Alteration of hormone transport	Heavy metals (mercury, lead, cadmium)	[13]
	Modification of hormone metabolism	Some pharmaceuticals (oral contraceptives, antiandrogens)	[14]

## References

[1] Tikariha R. (2023). Chemical Nature and Mechanism of Endocrine Disruptors (EDs): A Critical Review. Environmental Endocrine Toxicants.

[2] Warner G.R., Mourikes V.E., Neff A.M., Brehm E., Flaws J.A. (2020). Mechanisms of action of agrochemicals acting as endocrine disrupting chemicals. Mol. Cell. Endocrinol..

[3] Kabir E.R., Rahman M.S., Rahman I. (2015). A review on endocrine disruptors and their possible impacts on human health. Environ. Toxicol. Pharmacol..

[4] Ong H.-T., Samsudin H., Soto-Valdez H. (2022). Migration of endocrine-disrupting chemicals into food from plastic packaging materials: An overview of chemical risk assessment, techniques to monitor migration, and international regulations. Crit. Rev. Food Sci. Nutr..

[5] Plunk E.C., Richards S.M. (2020). Endocrine-disrupting air pollutants and their effects on the hypothalamus-pituitary-gonadal axis. Int. J. Mol. Sci..

[6] Panagopoulos P., Mavrogianni D., Christodoulaki C., Drakaki E., Chrelias G., Panagiotopoulos D., Potiris A., Drakakis P., Stavros S. (2023). Effects of endocrine disrupting compounds on female fertility. Best Pract. Res. Clin. Obstet. Gynaecol..

[7] Raja G.L., Subhashree K.D., Kantayya K.E. (2022). In utero exposure to endocrine disruptors and developmental neurotoxicity: Implications for behavioural and neurological disorders in adult life. Environ. Res..

[8] Domańska A., Orzechowski A., Litwiniuk A., Kalisz M., Bik W., Baranowska-Bik A. (2021). The beneficial role of natural endocrine disruptors: Phytoestrogens in Alzheimer’s disease. Oxidative Med. Cell. Longev..

[9] Maggio E.L., Zucca C., Grande M., Carrano R., Infante A., Bei R., Lucarini V., De Maio F., Focaccetti C., Palumbo C. (2024). Polyphenols Regulate the Activity of Endocrine-Disrupting Chemicals, Having Both Positive and Negative Effects. J. Xenobiotics.

[10] Araiza V.H., Segovia-Mendoza M., Castro K.E., Cruz S.M., Rueda K.C., de Leon C.T., Montor J.M. (2020). Bisphenol A: An endocrine-disruptor compound that modulates the immune response to infections. Front. Biosci.-Landmark.

[11] Burgos-Aceves M.A., Migliaccio V., Di Gregorio I., Paolella G., Lepretti M., Faggio C., Lionetti L. (2021). 1, 1, 1-trichloro-2, 2-bis (p-chlorophenyl)-ethane (DDT) and 1, 1-Dichloro-2, 2-bis (p, p’-chlorophenyl) ethylene (DDE) as endocrine disruptors in human and wildlife: A possible implication of mitochondria. Environ. Toxicol. Pharmacol..

[12] Sirohi D., Al Ramadhani R., Knibbs L.D. (2021). Environmental exposures to endocrine disrupting chemicals (EDCs) and their role in endometriosis: A systematic literature review. Rev. Environ. Health.

[13] Liu D., Shi Q., Liu C., Sun Q., Zeng X. (2023). Effects of endocrine-disrupting heavy metals on human health. Toxics.

[14] Wan M.L.Y., Co V.A., El-Nezami H. (2022). Endocrine disrupting chemicals and breast cancer: A systematic review of epidemiological studies. Crit. Rev. Food Sci. Nutr..

[15] Zhou Q., Chen J., Zhang J., Zhou F., Zhao J., Wei X., Zheng K., Wu J., Li B., Pan B. (2022). Toxicity and endocrine-disrupting potential of PM2.5: Association with particulate polycyclic aromatic hydrocarbons, phthalate esters, and heavy metals. Environ. Pollut..

[16] Bodziach K., Staniszewska M., Falkowska L., Nehring I., Ożarowska A., Zaniewicz G., Meissner W. (2021). Gastrointestinal and respiratory exposure of water birds to endocrine disrupting phenolic compounds. Sci. Total Environ..

[17] D’amico R., Di Paola D., Impellizzeri D., Genovese T., Fusco R., Peritore A.F., Gugliandolo E., Crupi R., Interdonato L., Cuzzocrea S. (2022). Chronic Exposure to Endocrine Disruptor Vinclozolin Leads to Lung Damage via Nrf2–Nf-kb Pathway Alterations. Int. J. Mol. Sci..

[18] Campolim C.M., Weissmann L., Ferreira C.K., Zordão O.P., Dornellas A.P., de Castro G., Zanotto T.M., Boico V.F., Quaresma P.G., Lima R.P. (2020). Short-term exposure to air pollution (PM2.5) induces hypothalamic inflammation, and long-term leads to leptin resistance and obesity via Tlr4/Ikbke in mice. Sci. Rep..

[19] Bolaji J.A. (2020). Indoor Environmental Irritant, Dibutyl Phthalate (DBP) and Sensory Irritation in the Airway: Contribution to Asthma Symptoms?.

[20] Casas M., Gascon M. (2020). Prenatal Exposure to Endocrine-Disrupting Chemicals and Asthma and Allergic Diseases. J. Investig. Allergol. Clin. Immunol..

[21] Zhang W., Wang Y., Wang L., Cao M., Cao H., Song M., Qian Y., Wang T., Liang Y., Jiang G. (2024). COPD-Like Phenotypes in TBC-Treated Mice Can be Effectively Alleviated via Estrogen Supplement. Environ. Sci. Technol..

[22] Adegoke E.O., Rahman M.S., Park Y.J., Kim Y.J., Pang M.G. (2021). Endocrine-disrupting chemicals and infectious diseases: From endocrine disruption to immunosuppression. Int. J. Mol. Sci..

[23] Mousavi S.E., Delgado-Saborit J.M., Adivi A., Pauwels S., Godderis L. (2022). Air pollution and endocrine disruptors induce human microbiome imbalances: A systematic review of recent evidence and possible biological mechanisms. Sci. Total Environ..

[24] Tang Z.-R., Xu X.-L., Deng S.-L., Lian Z.-X., Yu K. (2020). Oestrogenic endocrine disruptors in the placenta and the fetus. Int. J. Mol. Sci..

[25] Abellan A., Mensink-Bout S.M., Garcia-Esteban R., Beneito A., Chatzi L., Duarte-Salles T., Fernandez M.F., Garcia-Aymerich J., Granum B., Iñiguez C. (2022). In utero exposure to bisphenols and asthma, wheeze, and lung function in school-age children: A prospective meta-analysis of 8 European birth cohorts. Environ. Int..

[26] Guarnotta V., Amodei R., Frasca F., Aversa A., Giordano C. (2022). Impact of chemical endocrine disruptors and hormone modulators on the endocrine system. Int. J. Mol. Sci..

[27] Kirtana A., Seetharaman B. (2022). Comprehending the role of endocrine disruptors in inducing epigenetic toxicity. Endocr. Metab. Immune Disord. Drug Targets Former. Curr. Drug Targets-Immune Endocr. Metab. Disord..

[28] Basak S., Das M.K., Duttaroy A.K. (2020). Plastics derived endocrine-disrupting compounds and their effects on early development. Birth Defects Res..

[29] Buoso E., Masi M., Racchi M., Corsini E. (2020). Endocrine-disrupting chemicals’(EDCs) effects on tumour microenvironment and cancer progression: Emerging contribution of RACK1. Int. J. Mol. Sci..

[30] Sharma D., Bhartiya D. (2022). Dysfunctional ovarian stem cells due to neonatal endocrine disruption result in PCOS and ovarian insufficiency in adult mice. Stem Cell Rev. Rep..

[31] Palacios-Arreola M.I., Moreno-Mendoza N.A., Nava-Castro K.E., Segovia-Mendoza M., Perez-Torres A., Garay-Canales C.A., Morales-Montor J. (2022). The endocrine disruptor compound bisphenol-A (BPA) regulates the intra-tumoral immune microenvironment and increases lung metastasis in an experimental model of breast cancer. Int. J. Mol. Sci..

[32] Wu M., Wang S., Weng Q., Chen H., Shen J., Li Z., Wu Y., Zhao Y., Li M., Wu Y. (2021). Prenatal and postnatal exposure to Bisphenol A and Asthma: A systemic review and meta-analysis. J. Thorac. Dis..

[33] Boissiere-O’neill T., Lee W.R., Blake T.L., Sly P.D., Vilcins D. (2023). Exposure to endocrine-disrupting plasticisers and lung function in children and adolescents: A systematic review and meta-analysis. Environ. Res..

[34] Shah R. (2020). Pesticides and human health. Emerging Contaminants.

[35] Dodson R.E., Nishioka M., Standley L.J., Perovich L.J., Brody J.G., Rudel R.A. (2012). Endocrine disruptors and asthma-associated chemicals in consumer products. Environ. Health Perspect..

[36] Xu J., Xu L., Sui P., Chen J., Moya E.A., Hume P., Janssen W.J., Duran J.M., Thistlethwaite P., Carlin A. (2022). Excess neuropeptides in lung signal through endothelial cells to impair gas exchange. Dev. Cell.

[37] Loffredo L.F., Coden M.E., Berdnikovs S. (2020). Endocrine disruptor bisphenol A (BPA) triggers systemic para-inflammation and is sufficient to induce airway allergic sensitization in mice. Nutrients.

[38] Brassea-Pérez E., Hernández-Camacho C.J., Labrada-Martagón V., Vázquez-Medina J.P., Gaxiola-Robles R., Zenteno-Savín T. (2022). Oxidative stress induced by phthalates in mammals: State of the art and potential biomarkers. Environ. Res..

[39] Lee G.H., Jin S.W., Choi J.H., Han E.H., Hwang Y.P., Choi C.Y., Jeong H.G. (2021). Influence of o, p′-DDT on MUC5AC expression via regulation of NF-κB/AP-1 activation in human lung epithelial cells. J. Toxicol. Environ. Health Part A.

[40] Hoang T.T., Qi C., Paul K.C., Lee M., White J.D., Richards M., Auerbach S.S., Long S., Shrestha S., Wang T. (2021). Epigenome-wide DNA methylation and pesticide use in the agricultural lung health study. Environ. Health Perspect..

[41] Zhou Z., Goodrich J.M., Strakovsky R.S. (2020). Mitochondrial epigenetics and environmental health: Making a case for endocrine disrupting chemicals. Toxicol. Sci..

[42] Reddam A., McLarnan S., Kupsco A. (2022). Environmental chemical exposures and mitochondrial dysfunction: A review of recent literature. Curr. Environ. Health Rep..

[43] Wang L., Luo D., Liu X., Zhu J., Wang F., Li B., Li L. (2021). Effects of PM2.5 exposure on reproductive system and its mechanisms. Chemosphere.

[44] Quoc Q.L., Cao T.B.T., Kim S.-H., Choi Y., Ryu M.S., Choi Y., Park H.-S., Shin Y.S. (2023). Endocrine-disrupting chemical exposure augments neutrophilic inflammation in severe asthma through the autophagy pathway. Food Chem. Toxicol..

[45] Migliaccio S., Bimonte V.M., Besharat Z.M., Sabato C., Lenzi A., Crescioli C., Ferretti E. (2021). Environmental contaminants acting as endocrine disruptors modulate atherogenic processes: New risk factors for cardiovascular diseases in women?. Biomolecules.

[46] Wang J., Ma T., Ma D., Li H., Hua L.M., He Q., Deng X. (2021). The impact of air pollution on neurodegenerative diseases. Ther. Drug Monit..

[47] Celen H., Dens A.-C., Ronsmans S., Michiels S., De Langhe E. (2022). Airborne pollutants as potential triggers of systemic autoimmune rheumatic diseases: A narrative review. Acta Clin. Belg..

[48] Ghassabian A., Vandenberg L., Kannan K., Trasande L. (2022). Endocrine-disrupting chemicals and child health. Annu. Rev. Pharmacol. Toxicol..

[49] Yanagisawa R., Koike E., Win-Shwe T.-T., Takano H. (2022). Effects of oral exposure to low-dose Bisphenol S on allergic asthma in mice. Int. J. Mol. Sci..

[50] Quoc Q.L., Bich T.C.T., Kim S.-H., Ryu M.S., Park H.-S., Shin Y.S. (2022). Mono-n-butyl phthalate regulates nuclear factor erythroid 2–related factor 2 and nuclear factor kappa B pathway in an ovalbumin-induced asthma mouse model. Food Chem. Toxicol..

[51] Ramírez V., Robles-Aguilera V., Salcedo-Bellido I., Gálvez-Ontiveros Y., Rodrigo L., Martinez-Gonzalez L.J., Monteagudo C., Álvarez-Cubero M.J., Rivas A. (2022). Effects of genetic polymorphisms in body mass index according to dietary exposure to bisphenols and parabens. Chemosphere.

[52] Erden E.S., Motor S., Ustun I., Demirköse M., Yuksel R., Okur R., Oktar S., Yakar Y., Sungur S., Gokce C. (2014). Investigation of Bisphenol A as an endocrine disruptor, total thiol, malondialdehyde, and C-reactive protein levels in chronic obstructive pulmonary disease. Eur. Rev. Med. Pharmacol. Sci..

[53] Xing J., Zhang S., Zhang M., Hou J. (2022). A critical review of presence, removal and potential impacts of endocrine disruptors bisphenol A. Comp. Biochem. Physiol. Part C Toxicol. Pharmacol..

[54] Faheem N.M., El Askary A., Gharib A.F. (2021). Lycopene attenuates bisphenol A–induced lung injury in adult albino rats: A histological and biochemical study. Environ. Sci. Pollut. Res..

[55] Voynow J.A., Shinbashi M. (2021). Neutrophil elastase and chronic lung disease. Biomolecules.

[56] Wooding D.J., Ryu M.H., Li H., Alexis N.E., Pena O., Carlsten C. (2020). Acute air pollution exposure alters neutrophils in never-smokers and at-risk humans. Eur. Respir. J..

[57] Rosyid A.N., Saputra P.B.T., Purwati D.D., Ulhaq A.U.D., Yolanda S., Djatioetomo Y.C.E.D., Bakhtiar A. (2023). Neutrophil Elastase in the Pathogenesis of Chronic Obstructive Pulmonary Disease: A Review. Curr. Respir. Med. Rev..

[58] Fang L., Sun Q., Roth M. (2020). Immunologic and non-immunologic mechanisms leading to airway remodeling in asthma. Int. J. Mol. Sci..

[59] Wang W.-J., Peng K., Lu X., Zhu Y.-Y., Li Z., Qian Q.-H., Yao Y.-X., Fu L., Wang Y., Huang Y.-C. (2023). Long-term cadmium exposure induces chronic obstructive pulmonary disease-like lung lesions in a mouse model. Sci. Total Environ..

[60] Li Q., Sun J., Chen X., Li S., Wang Y., Xu C., Zhao J., Zhu Z., Tian L. (2020). Integrative characterization of fine particulate matter-induced chronic obstructive pulmonary disease in mice. Sci. Total Environ..

[61] Birnbaum L.S., Fenton S.E. (2003). Cancer and developmental exposure to endocrine disruptors. Environ. Health Perspect..

[62] Calaf G.M., Ponce-Cusi R., Aguayo F., Bleak T.C., Muñoz J.P. (2020). Endocrine disruptors from the environment affecting breast cancer. Oncol. Lett..

[63] Jiang C.-L., He S.-W., Zhang Y.-D., Duan H.-X., Huang T., Huang Y.-C., Li G.-F., Wang P., Ma L.-J., Zhou G.-B. (2017). Air pollution and DNA methylation alterations in lung cancer: A systematic and comparative study. Oncotarget.

[64] Xue Y., Wang L., Zhang Y., Zhao Y., Liu Y. (2022). Air pollution: A culprit of lung cancer. J. Hazard. Mater..

[65] Ji S.M., Choi J.-S., Lee J.Y., Kim S., Bae W.-Y., Jang Y.W., Kim J.-E., Lee S.H., Nam S., Jeong J.-W. (2023). Mild exposure to fine particulate matter promotes angiogenesis in non-small cell lung carcinoma. Environ. Pollut..

[66] Asanov M., Bonassi S., Proietti S., Minina V.I., Tomino C., El-Zein R. (2021). Genomic instability in chronic obstructive pulmonary disease and lung cancer: A systematic review and meta-analysis of studies using the micronucleus assay. Mutat. Res. Rev. Mutat. Res..

[67] Attafi I.M., Bakheet S.A., Korashy H.M. (2020). The role of NF-κB and AhR transcription factors in lead-induced lung toxicity in human lung cancer A549 cells. Toxicol. Mech. Methods.

[68] D’amico R., Monaco F., Fusco R., Siracusa R., Impellizzeri D., Peritore A.F., Crupi R., Gugliandolo E., Cuzzocrea S., Di Paola R. (2021). Atrazine inhalation worsen pulmonary fibrosis regulating the nuclear factor-erythroid 2-related factor (Nrf2) pathways inducing brain comorbidities. Cell. Physiol. Biochem.

[69] Genovese T., Duranti A., Monaco F., Siracusa R., Fusco R., Impellizzeri D., D’Amico R., Cordaro M., Cuzzocrea S., Di Paola R. (2023). Inhibition of Fatty Acid Amide Hydrolase (FAAH) Regulates NF-kb Pathways Reducing Bleomycin-Induced Chronic Lung Inflammation and Pulmonary Fibrosis. Int. J. Mol. Sci..

[70] Siswanto S., Wardhani B.W. (2022). Association of Environmental Pollutants Exposure with Pulmonary Fibrosis: A Mini Review of Molecular Mechanism Mediated. Pharm. Sci. Res..

[71] Xiao T., Zou Z., Xue J., Syed B.M., Sun J., Dai X., Shi M., Li J., Wei S., Tang H. (2021). LncRNA H19-mediated M2 polarization of macrophages promotes myofibroblast differentiation in pulmonary fibrosis induced by arsenic exposure. Environ. Pollut..

[72] Majewski S., Piotrowski W.J. (2020). Air pollution—An overlooked risk factor for idiopathic pulmonary fibrosis. J. Clin. Med..

